# Cohort Profile Update: Born in Bradford

**DOI:** 10.1093/ije/dyae037

**Published:** 2024-03-29

**Authors:** Rosemary R C McEachan, Gillian Santorelli, Aidan Watmuff, Dan Mason, Sally E Barber, Daniel D Bingham, Philippa K Bird, Laura Lennon, Dan Lewer, Mark Mon-Williams, Katy A Shire, Dagmar Waiblinger, Jane West, Tiffany C Yang, Deborah A Lawlor, Kate E Pickett, John Wright

**Affiliations:** Bradford Institute for Health Research, Bradford Teaching Hospitals NHS Foundation Trust, Bradford, West Yorkshire, UK; Bradford Institute for Health Research, Bradford Teaching Hospitals NHS Foundation Trust, Bradford, West Yorkshire, UK; Bradford Institute for Health Research, Bradford Teaching Hospitals NHS Foundation Trust, Bradford, West Yorkshire, UK; Bradford Institute for Health Research, Bradford Teaching Hospitals NHS Foundation Trust, Bradford, West Yorkshire, UK; Bradford Institute for Health Research, Bradford Teaching Hospitals NHS Foundation Trust, Bradford, West Yorkshire, UK; Faculty of Health Studies, University of Bradford, Bradford, West Yorkshire, UK; Bradford Institute for Health Research, Bradford Teaching Hospitals NHS Foundation Trust, Bradford, West Yorkshire, UK; Bradford Institute for Health Research, Bradford Teaching Hospitals NHS Foundation Trust, Bradford, West Yorkshire, UK; Bradford Institute for Health Research, Bradford Teaching Hospitals NHS Foundation Trust, Bradford, West Yorkshire, UK; School of Psychology, University of Leeds, Leeds, West Yorkshire, UK; Bradford Institute for Health Research, Bradford Teaching Hospitals NHS Foundation Trust, Bradford, West Yorkshire, UK; Bradford Institute for Health Research, Bradford Teaching Hospitals NHS Foundation Trust, Bradford, West Yorkshire, UK; Bradford Institute for Health Research, Bradford Teaching Hospitals NHS Foundation Trust, Bradford, West Yorkshire, UK; Bradford Institute for Health Research, Bradford Teaching Hospitals NHS Foundation Trust, Bradford, West Yorkshire, UK; MRC Integrative Epidemiology Unit, University of Bristol, Clifton, Bristol, UK; Bristol Medical School, University of Bristol, Clifton, Bristol, UK; Department of Health Sciences, University of York, York, West Yorkshire, UK; Bradford Institute for Health Research, Bradford Teaching Hospitals NHS Foundation Trust, Bradford, West Yorkshire, UK

Key Features

- Born in Bradford (BiB) is a multi-ethnic birth cohort in Bradford, UK, which recruited 12 453 women with 13 776 pregnancies and 3448 of their partners between 2007 and 2011.
- Routine health and education data have been collected since birth and cohort subsamples have been followed up to age 5 years. Metabolomics and genetic data are available for ∼11 000 mothers and ∼9000 children. The cohort works closely with communities and health and education providers using findings to influence policy.
- This update summarizes the most recent follow-up in school and community settings when children were aged 6–11 years and key findings from the cohort.
- Extensive new data for BiB families have been collected, including questionnaires, cognitive and sensorimotor development, built and natural environment measures, biological samples, with continued record linkage.
- Measures of wellbeing and cognitive/sensorimotor development were extended to include non-BiB children in a whole-school recruitment approach.
- Applications to use data are invited via our website (www.borninbradford.nhs.uk).

## The original cohort

The Born in Bradford (BiB) cohort is based in Bradford—a large city in the north of England in the UK. The aim of the cohort is to explore why some families stay healthy whilst others fall ill and to use this information to develop and evaluate interventions and improve services within the city.

The cohort takes a broad socio-ecological perspective on health, focusing on a range of determinants that include genetic, multi-omic, lifestyle, interpersonal, community, organizational and environmental factors. BiB works closely with communities and stakeholders to co-produce research priorities^1^ and provides a model for the translation of research into practice.

Baseline recruitment occurred between 2007 and 2011.^2^ Women and their partners were recruited between 2007 and 2011 when the women attended a routine antenatal clinic appointment at the city’s main maternity unit. Around 83% of pregnant women attended this appointment and, of these, >80% were recruited. The cohort recruited 12 453 women with 13 776 pregnancies (recruited at ∼26 weeks’ gestation) and 3448 partners.

At time of recruitment, half (49%) of the cohort were of South Asian heritage and 68% of the children lived in the most deprived quintile of neighbourhoods in England and Wales, representative of the wider population.^2^ Since baseline repeat assessments have been conducted on subsamples including a focus on obesity (with repeat data collection for 1763 children at 6 months, 12 months, 18 months, 2 years and 3 years), allergies and infections (2594 children at 12 months, 2 years and 4 years) and cognitive development (3444 children at 5 years). Routine health data linkage is available for 98% of participants and education data linked for 85% of children. Stored bio-samples include pregnancy blood (*N* = 11 625) and urine (*N* = 6996) and cord blood (*N* = 9303). Exome sequencing is available for 10 531 mothers and 9158 children, and metabolomics are available from 11 479 pregnancy samples, 7890 cord bloods and 2108 children’s samples at age 2 years. A summary of available data can be found in Supplementary Figure S1, available as Supplementary data at *IJE* online.

## What is the reason for the new data collection?

The aim was to assess the health and life circumstances of families when children were aged 6–11 years. By collecting data on a broad range of topics, we aimed to provide a resource to allow exploration of the determinants of children’s pre-pubertal health and development, including through understanding parents’ health and wellbeing. We also wished to obtain data on exposures in childhood that might influence future health.^3^ We aimed to include BiB children and their peers in a whole-population approach to have maximum policy relevance for partners in the city. In this update, we summarize new findings and data available since the original cohort profile^2^ from (i) the follow-up of cohort children aged 6–11 years and their peers in primary school and cohort children and parents in community settings; (ii) measures of the built and natural environment calculated for geocoded home addresses for BiB families; (iii) assays of stored blood samples, including metabolomics and proteomics; (iv) extensive health and education record linkage data.

## What will be the new areas of research?

We focus on health inequalities, particularly variations in outcomes by ethnic groups. The three priority research areas for the new data collection included: social and emotional wellbeing; growth, adiposity and cardiometabolic measurements; and child cognitive and sensorimotor function. During the COVID-19 pandemic, the cohort used an adaptive, mixed-methods research protocol to capture important information on the impacts of the pandemic on health and wellbeing.^4^

## Who is in the cohort?

For our new data collection, we recruited participants through three study arms:

- In the ‘BiB Growing Up’ study arm, BiB parents and children completed measures either at home or at community or clinic appointments, including in a mobile health research unit with dual-energy X-ray absorptiometry scanning capability. At these appointments, they completed questionnaire and physical measurements, including blood sampling. Parents gave consent for their own and their child’s participation.
- ‘School Nurse Measurements’ within primary schools collected BiB children’s blood, accelerometry and physical measurements. Parents gave opt-out consent for measurements and opt-in consent for blood draws and accelerometry.
- In the ‘Primary School Years’ study arm, whole-school classroom-based assessments collected BiB and non-BiB child-reported outcomes and cognitive/sensorimotor. Head teachers gave opt-in consent for the participation of their school and parents gave opt-out consent for their child to take part in assessments.

Parents were assessed only in the Growing Up study arm. BiB children could participate in the Growing Up, School Nurse Measurements and Primary School Years study arms. Non-BiB children could only participate in the Primary School Years study arm. Further details about recruitment and consent are reported elsewhere^3^.

Recruitment for Primary School Years took place between 16 May 2016 and 10 July 2019. We approached 90 primary schools that had high numbers of BiB children attending; 89 schools participated. The Growing Up study and school nurse measurements commenced on 23 February 2017 and were paused on 20 March 2020 due to lockdowns and school closures imposed in the UK as a result of the pandemic. Further recruitment was undertaken between 10 April and 24 June 2020 for urgent COVID-19-related research projects. These families provided consent to routine data linkage but completed different surveys to the full Growing Up sample.

**Figure 1.**
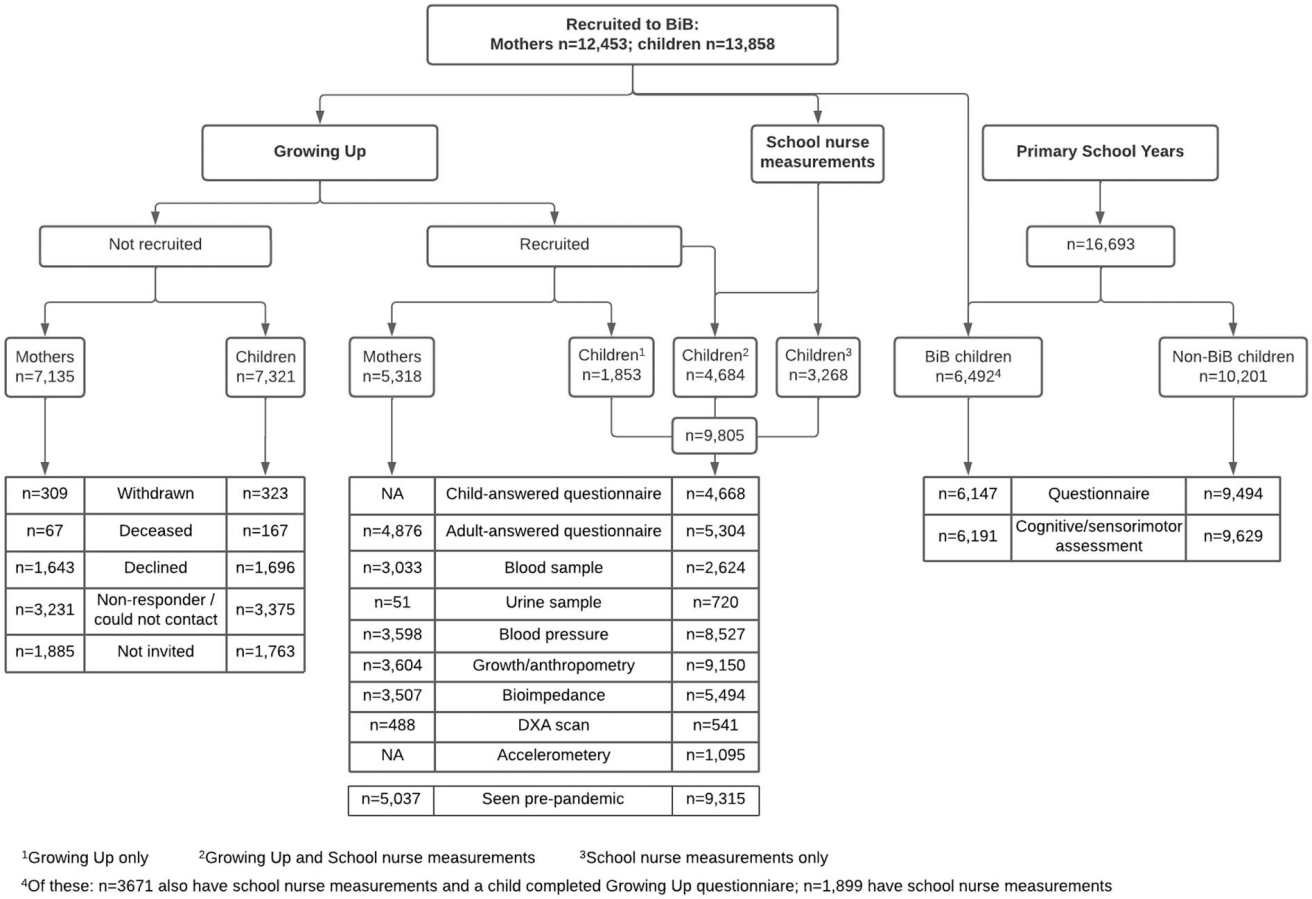
Overview of recruitment and data collection activities for the new Born in Bradford data collection at age 6–11 years

In total, 5318 BiB mothers [mean age at follow-up 37.9 (SD 5.6) years], 9805 BiB children [mean age 9.2 (SD 1.1) years] and 838 fathers and partners [mean age 38.9 (SD 5.4) years] were recruited. This represents 43%, 74% and 25% of the original cohort, respectively. In addition, 10 201 non-BiB children participated in the Primary School Years study. Figure 1 summarizes mothers and children recruited via the different recruitment channels. Table 1 compares the characteristics of mothers recruited to the BiB Growing Up study with the original cohort. This shows that the sample is representative of the original cohort in terms of deprivation level, but with over-representation of mothers of South Asian origin (61.7% compared with 49.0%) and mothers born outside the UK (51.6% compared with 46.3%).

**Illustration 1.**
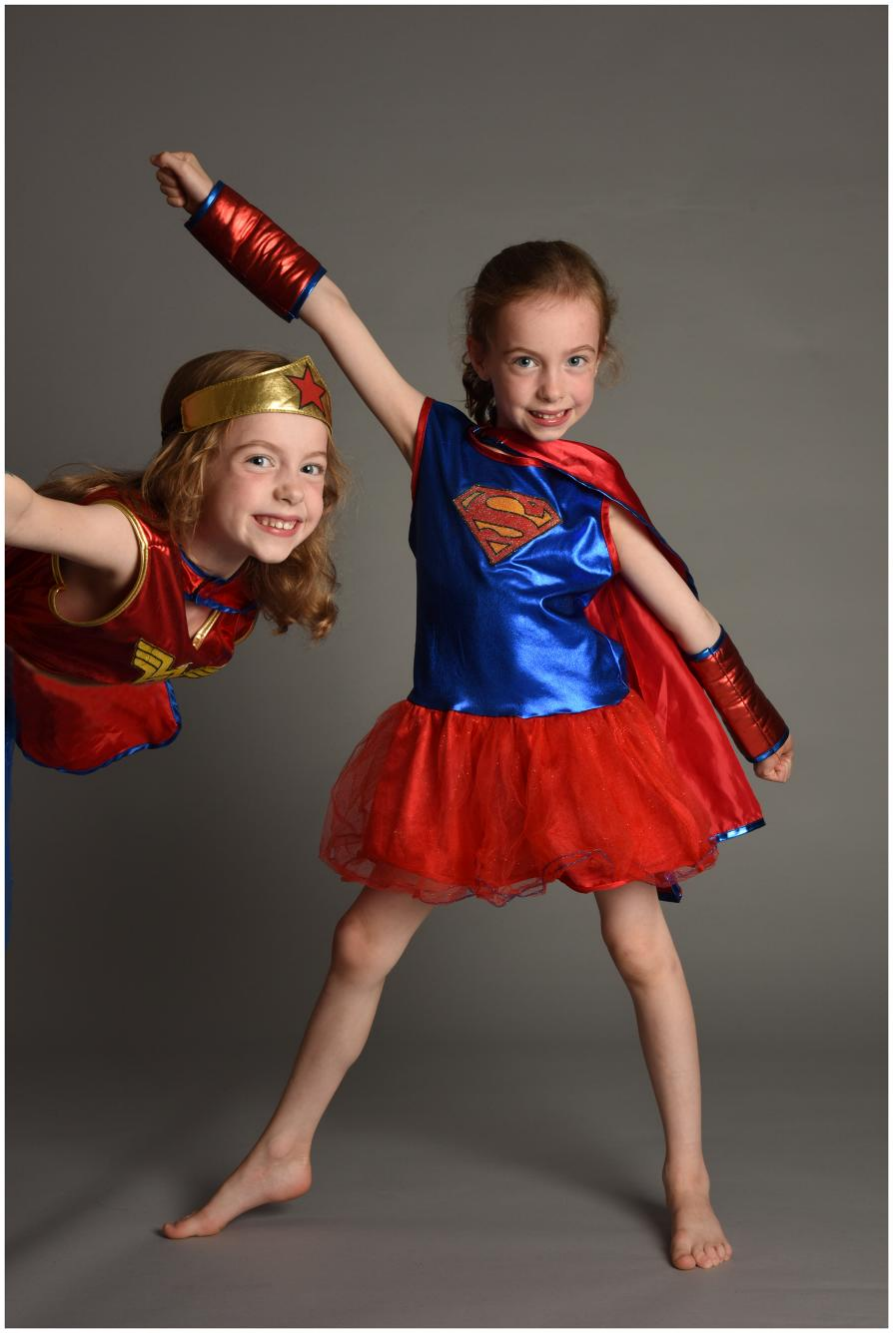
BiB participants Aoife and Ciara (aged 6 years). Photo by Ian Beesley; from the Born in Bradford website, reproduced with permission

**Illustration 2.**
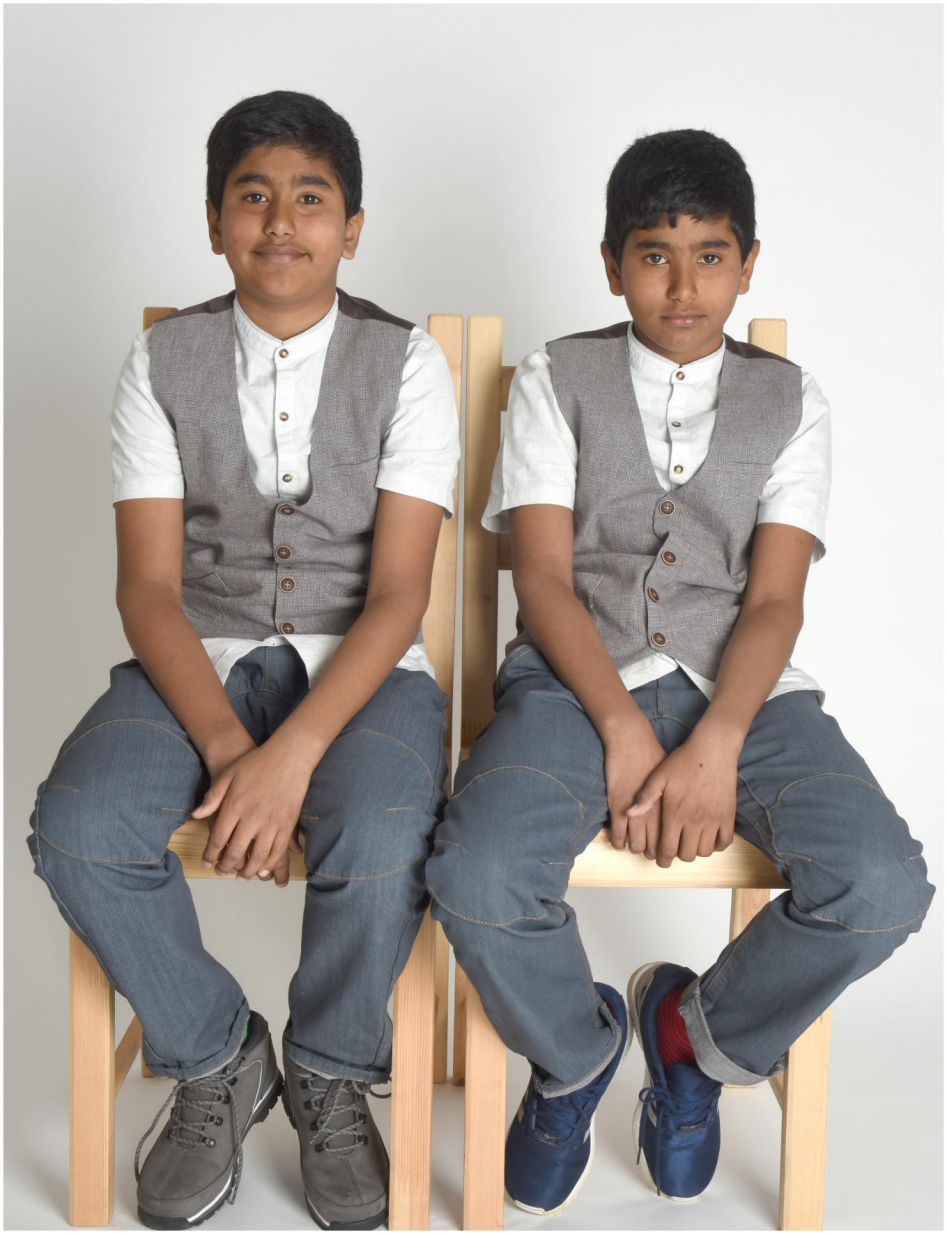
BiB participants Darwood and Ishaaq (aged 10 years). Photo by Ian Beesley; from the Born in Bradford website, reproduced with permission

**Table 1.** Comparison of Born in Bradford Growing Up cohort with baseline Born in Bradford cohort

			Baseline Born in Bradford cohort		Born in Bradford Growing Up cohort	
Mothers recruited			12 453	NA	5318	NA
Resident in Bradford district						
	No		385	3.1%	57	1.1%
	Yes		11 467	92.1%	5012	94.2%
IMD quintile^a^						
	1 (most deprived)		7904	63.5%	3479	65.4%
	2		2126	17.1%	890	16.7%
	3		1249	10.0%	506	9.5%
	4		355	2.9%	123	2.3%
	5 (least deprived)		215	1.7%	71	1.3%
Ethnic group						
	South Asian		6102	49.0%	3279	61.7%
		Pakistani heritage^b^	5297	86.8%	2885	88.0%
	White		4976	40.0%	1589	29.9%
		White British^c^	4584	92.1%	1467	92.3%
	Mixed		256	2.1%	83	1.6%
	Black		279	2.2%	75	1.4%
	Other		288	2.3%	117	2.2%
Born in the UK						
	No		5781	46.4%	2734	51.4%
	Yes		6672	53.6%	2584	48.6%

IMD, Index of multiple deprivation.

Values are *n* (%).

a IMD for address at BiB recruitment, based on 2010 scores.

b Percentage reflects Pakistani heritage as a proportion of South Asian participants.

c Percentage reflects White British as a proportion of White participants.

## What has been measured?

Table 2 summarizes the key data collected by study arm; further detail is available in our protocol^3^ and via our data dictionaries accessible via our website (www.borninbradford.nhs.uk). Indicators relating to the built environment, including air quality, green space access and local food environment, have been calculated at an address level and linked to the data set.^5^ Child-completed cognitive and sensorimotor assessments are available for BiB and non-BiB participants.^6^ Routine primary and secondary health data and education data are collected through record linkage. Metabolomics, which have already been profiled for pregnancy and early-life exposures,^7^ have been repeated for newly collected samples. Blood samples have been collected from 3033 mothers and 2624 children and accelerometry data have been collected for 1095 children.

**Table 2.** Overview of new data collection at age 6–11 years

Data		Growing Up	School nurse measures	Primary School Years
Parents				
	Parent questionnaire (time: 40 min. Includes demographics, home and neighbourhood; socio-economic circumstances; parent health and health behaviours; physical activity and sedentary behaviour; child health and development; socio-emotional wellbeing; acculturation; diet^a^; parenting^a^; child allergies^b^)	X		
	Parent-reported strengths and difficulties questionnaire	X		
	Height and weight	X		
	Subscapular and triceps skinfold	X		
	Waist circumference	X		
	Bioimpedance	X		
	Blood pressure and pulse rate	X		
	Blood sample	X		
	Dual X-ray absorptiometry (DEXA) scan	X		
Children				
	‘Me and my life’ questionnaire (time: 15 min. Includes happiness and health; material wellbeing; family, friends and bullying; school; neighbourhood; demographics; aspirations and acculturation)			X
	Diet and activity questionnaire (time: 30 min. Includes physical activity and sedentary behaviour, determinants of physical activity, diet)	X	X	
	Child-completed computerized cognitive and sensorimotor assessment (time: 30 min)			X
	Height and weight	X	X	
	Subscapular and triceps skinfold	X	X	
	Waist circumference	X		
	Bioimpedance	X	X	
	Blood pressure and pulse rate	X	X	
	Accelerometry		X	
	Blood sample	X	X	
	Urine sample^c^	X		
	DEXA scan			
Teacher				
	Teacher-reported strengths and difficulties questionnaire (child behaviour)			X

Data only collected for:

a families followed up previously as part of obesity-related sub-study.

b families followed up previously as part of allergy and infection sub-sample.

c families who had taken part in renal sub-study.

A particular strength of the cohort is the large numbers of South Asian and White European participants, which provides power to explore differences in health between these two groups, and the inclusion of families living in deprived areas who are typically under-represented in longitudinal studies.

Tables 3 and 4 detail the breadth of information collected, stratifying responses by White European, South Asian or other ethnic origin, highlighting key differences in our sample. Supplementary Tables S1 and S2 (available as Supplementary data at *IJE* online) present further key characteristics using more granular ethnic origin groupings.

**Table 3.** Characteristics of mothers in the Born in Bradford Growing Up study by ethnic group

Characteristic		Total sample (*n *=* *5318, 100.0%)		South Asian (*n *=* *3279, 61.7%)		White (*n *=* *1589, 29.9%)		Other (*n *=* *275, 5.2%)	
Mother’s age (years)		37.9	5.6	38.0	5.2	37.6	6.2	39.0	6.0
**Growing Up adult survey** ^a^		**4876**	**100%**	**2985**	**61.2%**	**1484**	**30.4%**	**247**	**5.1%**
Relationship status									
	Married and living with partner	3781	77.5%	2736	91.7%	769	51.8%	162	65.6%
	Not married and living with partner	385	7.9%	8	0.3%	343	23.1%	17	6.9%
	Not living with partner	694	14.2%	232	7.8%	368	24.8%	65	26.3%
Housing occupation status									
	Owns/with mortgage	3235	66.3%	2201	73.7%	833	56.1%	108	43.7%
	Lives rent free	273	5.6%	238	8.0%	26	1.8%	5	2.0%
	Rents	1306	26.8%	517	17.3%	603	40.6%	125	50.6%
	Other/don’t know	19	0.4%	3	0.1%	12	0.8%	3	1.2%
Number of occupants in household									
	1–3	571	11.7%	155	5.2%	338	22.8%	58	23.5%
	4–5	2255	46.2%	1167	39.1%	883	59.5%	120	48.6%
	6–7	1265	25.9%	1063	35.6%	124	8.4%	42	17.0%
	8+	432	8.9%	393	13.2%	24	1.6%	3	1.2%
Participant’s employment status									
	Unemployed	2592	53.2%	2055	68.8%	376	25.3%	80	32.4%
	Employed	2267	46.5%	919	30.8%	1105	74.5%	164	66.4%
Partner’s employment status									
	Unemployed	417	8.6%	265	8.9%	122	8.2%	19	7.7%
	Employed	3808	78.1%	2478	83.0%	1043	70.3%	163	66.0%
	No partner	552	11.3%	206	6.9%	269	18.1%	56	22.7%
Household’s current financial situation									
	Financially secure	3285	67.4%	1987	66.6%	1033	69.6%	155	62.8%
	Financially insecure	1568	32.2%	989	33.1%	440	29.6%	89	36.0%
Household’s financial situation compared with 1 year ago									
	Better	1006	20.6%	534	17.9%	378	25.5%	63	25.5%
	Worse	827	17.0%	452	15.1%	300	20.2%	49	19.8%
	Same	2888	59.2%	1889	63.3%	777	52.4%	125	50.6%
	Do not wish to answer	132	2.7%	102	3.4%	17	1.1%	7	2.8%
Does participant think that people can be trusted?									
	Can be trusted	1865	38.2%	1003	33.6%	711	47.9%	89	36.0%
	Can’t be too careful	2990	61.3%	1969	66.0%	767	51.7%	156	63.2%
Self-rated general health									
	Excellent	317	6.5%	206	6.9%	83	5.6%	21	8.5%
	Very good	960	19.7%	475	15.9%	412	27.8%	48	19.4%
	Good	2294	47.0%	1442	48.3%	649	43.7%	123	49.8%
	Fair	973	20.0%	644	21.6%	261	17.6%	37	15.0%
	Poor	320	6.6%	213	7.1%	73	4.9%	17	6.9%
Self-rated dental health									
	Excellent	374	7.7%	215	7.2%	127	8.6%	23	9.3%
	Very good	1007	20.7%	518	17.4%	417	28.1%	51	20.6%
	Good	2211	45.3%	1443	48.3%	563	37.9%	116	47.0%
	Fair	892	18.3%	574	19.2%	256	17.3%	34	13.8%
	Poor	380	7.8%	230	7.7%	115	7.7%	22	8.9%
Presence of long-term health condition									
	No	3699	75.9%	2351	78.8%	1051	70.8%	186	75.3%
	Yes	1056	21.7%	557	18.7%	401	27.0%	53	21.5%
Measure of current depression^b^									
	No significant symptoms	3349	68.7%	2147	71.9%	912	61.5%	171	69.2%
	Mild symptoms	889	18.2%	507	17.0%	314	21.2%	48	19.4%
	Moderate symptoms	322	6.6%	186	6.2%	111	7.5%	12	4.9%
	Moderately severe symptoms	165	3.4%	84	2.8%	71	4.8%	6	2.4%
	Severe symptoms	72	1.5%	38	1.3%	26	1.8%	7	2.8%
Measure of generalized anxiety^c^									
	Minimal	3568	73.2%	2279	76.3%	980	66.0%	189	76.5%
	Mild	718	14.7%	411	13.8%	254	17.1%	32	13.0%
	Moderate	283	5.8%	160	5.4%	105	7.1%	11	4.5%
	Severe	209	4.3%	114	3.8%	76	5.1%	12	4.9%
Smoking status									
	Never smoked	3677	75.4%	2757	92.4%	641	43.2%	175	70.9%
	Previously smoked	562	11.5%	77	2.6%	428	28.8%	35	14.2%
	Currently smoke	536	11.0%	128	4.3%	348	23.5%	32	13.0%
Alcohol consumption status									
	Does not drink alcohol	3643	74.7%	2908	97.4%	461	31.1%	167	67.6%
	Does drink alcohol	1084	22.2%	23	0.8%	940	63.3%	75	30.4%
	Do not wish to answer	32	0.7%	8	0.3%	20	1.3%	2	0.8%
Physical activity: metabolic equivalent of task category^d^									
	Inactive	88	1.8%	68	2.3%	17	1.1%	1	0.4%
	Minimally active	2392	49.1%	1593	53.4%	595	40.1%	128	51.8%
	Active	1802	37.0%	946	31.7%	697	47.0%	98	39.7%
Body mass index		28.5	6.2	28.6	5.8	28.4	6.8	28.9	6.4
**Healthcare data** ^e^		**4970**	**100%**	**3085**	**62.1%**	**1471**	**29.6%**	**249**	**5%**
Number of general practitioner attendances		5	3.0–10.0	5	3.0–10.0	5	2.0–8.0	6	3.0–10.0
Number of prescriptions issued		6	3.0–16.0	7	3.0–16.0	6	2.0–14.0	6	3.0–15.0

Values are *n* (%), mean (standard deviation) or median (interquartile range). For brevity, missing values have been calculated but not reported and some categories have been collapsed. Refer to Supplementary Table S1 (available as Supplementary data at *IJE* online) for full table with missing values displayed.

a Sample is mothers who have completed a Growing Up adult survey.

b Measured using the Patient Health Questionnaire (PHQ-8).^35^

c Measured using the Generalised Anxiety Disorder assessment (GAD-7).^36^

d Measured using the International Physical Activity Questionnaire (IPAQ)—short form.^37^

e Sample is mothers recruited to Growing Up with primary care data. Timescale is during the year prior to Growing Up recruitment.

**Table 4.** Characteristics of young people in the Born in Bradford Growing Up study arm by ethnic group

Characteristic		Total sample (*n *=* *6537, 100.0%)		South Asian (*n *=* *3894, 59.6%)		White (*n *=* *1974, 30.2%)		Other (*n *=* *661, 10.1%)	
Childs age (years)		9.3	1.1	9.2	1.0	9.4	1.1	9.4	1.1
Child’s general health									
	Poor	67	1.3%	49	1.5%	13	0.9%	5	1.0%
	Fair	343	6.5%	254	7.8%	68	4.5%	21	4.0%
	Good	1643	31.0%	1234	37.9%	262	17.2%	145	27.9%
	Very good	1585	29.9%	885	27.2%	518	34.0%	180	34.6%
	Excellent	1626	30.7%	809	24.9%	652	42.8%	164	31.5%
Child’s dental health									
	Poor	233	4.4%	184	5.7%	30	2.0%	19	3.7%
	Fair	496	9.4%	380	11.7%	81	5.3%	35	6.7%
	Good	1762	33.2%	1248	38.4%	353	23.1%	159	30.6%
	Very good	1323	24.9%	742	22.8%	433	28.4%	147	28.3%
	Excellent	1305	24.6%	645	19.8%	514	33.7%	146	28.1%
Strengths and difficulties questionnaire^a^									
	Prosocial score	9	8.0–10.0	10	8.0–10.0	9	7.0–10.0	9	8.0–10.0
	Total difficulties score (excluding prosocial)	8	4.0–12.0	7	4.0–11.0	8	4.0–14.0	8	5.0–12.0
**Physical activity questionnaire** ^b^		**4678**	**100%**	**2363**	**61.8%**	**1089**	**28.5%**	**368**	**9.6%**
Meets physical activity guidelines?^c^									
	Yes	2330	61.0%	1410	59.7%	699	64.2%	220	59.8%
	No	1492	39.0%	953	40.3%	390	35.8%	148	40.2%
**Biological measures** ^d^		**4826**	**100%**	**2855**	**59.2%**	**1502**	**31.1%**	**467**	**9.7%**
Child’s BMI category^e^									
	Underweight	152	3.1%	122	4.3%	22	1.5%	8	1.7%
	Healthy weight	3237	67.1%	1878	65.8%	1059	70.5%	299	64.0%
	Overweight	581	12.0%	325	11.4%	187	12.5%	69	14.8%
	Obese	856	17.7%	530	18.6%	234	15.6%	91	19.5%
**Healthcare data** ^f^		**4891**	**100%**	**3040**	**62.2%**	**1373**	**28.1%**	**473**	**9.7%**
Number of general practitioner attendances		2	1.0–4.0	2	1.0–4.0	2	1.0–3.0	2	1.0–4.0
Number of prescriptions issued		3	1.0–7.0	3	1.0–8.0	1	0.0–4.0	3	1.0–8.0

BMI, body mass index.

Values are *n* (%) or median (interquartile range). For brevity, missing values have been calculated but not reported. Refer to Supplementary Table S2 (available as Supplementary data at *IJE* online) for a full table with missing values displayed.

a Measured using strengths and difficulties questionnaire.^38^

b Sample is children who have a Growing Up child-completed survey.

c Calculated using the Physical Activity Questionnaire—Child (PAQ-C)^39^ and validated cut points.^40^

d Sample is children recruited to Growing Up with BMI measures.

e BMI calculated using UK90 reference table: underweight, z-score ≤–2.326; healthy weight, z-score >–2.326 and ≤1.036; overweight, z-score >1.036 and ≤1.645; and obese, z-score >1.645.

f Sample is children recruited to Growing Up with primary care data. Timescale is during the year prior to Growing Up recruitment.

Table 5 presents information from both BiB and non-BiB children recruited via our Primary School Years study arm.

**Table 5.** Wellbeing and availability of cognitive/sensorimotor assessments of young people participating in the Primary School Years study, overall and by Born in Bradford participation status

		Total sample		BiB participants		Non-BiB participants	
Completed wellbeing questionnaire (*n*)		15 641		6147		9494	
How often does your family get along well together?							
	Never	729	4.7%	258	4.2%	471	5.0%
	Some of the time	6845	43.8%	2701	43.9%	4144	43.6%
	All of the time	7689	49.2%	3050	49.6%	4639	48.9%
How often do you play in a park?							
	Never	1137	7.3%	429	7.0%	708	7.5%
	Sometimes	11 701	74.8%	4680	76.1%	7021	74.0%
	Very often	2386	15.3%	887	14.4%	1499	15.8%
Does your home have a garden where you can play?							
	No	2036	13.0%	770	12.5%	1266	13.3%
	Yes	13 357	85.4%	5294	86.1%	8063	84.9%
Do you have a park near your home where you can play with your friends?							
	No	4711	30.1%	1876	30.5%	2835	29.9%
	Yes	10 584	67.7%	4132	67.2%	6452	68.0%
Do you have a warm winter coat?							
	No	1402	9.0%	556	9.0%	846	8.9%
	Yes	12 594	80.5%	4964	80.8%	7630	80.4%
Do you have a computer, laptop or tablet with internet at home?							
	No	2733	17.5%	1023	16.6%	1710	18.0%
	Yes	11 563	73.9%	4614	75.1%	6949	73.2%
Do you have three meals every day?							
	No	2243	14.3%	894	14.5%	1349	14.2%
	Yes	11 891	76.0%	4656	75.7%	7235	76.2%
How often do you worry about how much money your family has?							
	Never	5264	33.7%	2128	34.6%	3136	33.0%
	Sometimes	6124	39.2%	2361	38.4%	3763	39.6%
	All of the time	4054	25.9%	1580	25.7%	2474	26.1%
How often do you feel happy?							
	Never	585	3.7%	230	3.7%	355	3.7%
	Some of the time	8306	53.1%	3292	53.6%	5014	52.8%
	All of the time	6420	41.0%	2495	40.6%	3925	41.3%
How often do you feel sad?							
	Never	3837	24.5%	1491	24.3%	2346	24.7%
	Some of the time	10 715	68.5%	4249	69.1%	6466	68.1%
	All of the time	863	5.5%	318	5.2%	545	5.7%
How often are you ill or unwell?							
	Never	2216	14.2%	816	13.3%	1400	14.7%
	Some of the time	11 966	76.5%	4757	77.4%	7209	75.9%
	All of the time	1247	8.0%	491	8.0%	756	8.0%
What do you do if you are worried about something?^a^							
	Keep it to myself	4828	30.9%	1880	30.6%	2948	31.1%
	Tell a friend	5192	33.2%	2033	33.1%	3159	33.3%
	Tell my mum/dad/guardian	11 616	74.3%	4623	75.2%	6993	73.7%
	Tell a teacher	7329	46.9%	2889	47.0%	4440	46.8%
When I find something really hard, I can work out what to do next							
	Never	1299	8.3%	450	7.3%	849	8.9%
	Some of the time	9429	60.3%	3759	61.2%	5670	59.7%
	All of the time	4409	28.2%	1767	28.7%	2642	27.8%
How many friends do you have?							
	Not many	2257	14.4%	877	14.3%	1380	14.5%
	Some	3647	23.3%	1397	22.7%	2250	23.7%
	Lots	9623	61.5%	3837	62.4%	5786	60.9%
How often do other children bully you?							
	Never	7276	46.5%	2882	46.9%	4394	46.3%
	Some of the time	6413	41.0%	2544	41.4%	3869	40.8%
	All of the time	1679	10.7%	625	10.2%	1054	11.1%
How often are you mean to other children at school?							
	Never	10 806	69.1%	4269	69.4%	6537	68.9%
	Some of the time	3645	23.3%	1431	23.3%	2214	23.3%
	All of the time	794	5.1%	312	5.1%	482	5.1%
How often do you feel left out of things by other children?							
	Never	5598	35.8%	2245	36.5%	3353	35.3%
	Some of the time	8129	52.0%	3200	52.1%	4929	51.9%
	All of the time	1729	11.1%	642	10.4%	1087	11.4%
How much do you like school?							
	I don’t like it	2032	13.0%	822	13.4%	1210	12.7%
	I like it a bit	4691	30.0%	1863	30.3%	2828	29.8%
	I like it a lot	8797	56.2%	3421	55.7%	5376	56.6%
Completed cognitive/sensorimotor assessments^b^		15 820	100.0%	6191	39.1%	9629	60.9%

Values are *n* (%). For brevity, missing values have been calculated but not reported.

a Values do not add up to 100% as questions allowed multiple responses.

b Further information on these measures is reported in^41^.

## What has it found? Key findings and publications

BiB data, including that collected prior to this recent follow-up, have been used in >200 publications and findings have been used to inform policy and practice internationally. Research briefings are available on our website https://borninbradford.nhs.uk/our-findings/different-findings-in-a-nutshell/.

### Gene function and loss-of-function variants

Exome sequencing in the BiB cohort has identified >1100 naturally occurring gene knockouts (homozygous loss-of-function variants) in 820 consanguineous participants.^8^ This research has led to developments in drug discovery in primary hyperoxaluria^9^ and psoriasis.^10^

### Congenital anomalies

Of the Pakistani-origin parents in BiB, 37% were in first-cousin marriages. The risk of congenital anomalies was doubled (from 3% to 6%) in first-cousin marriages and explained 30% of the genetic disorders observed. Similar results were found in relation to maternal age, with infants of women over the age of 34 years having a 4% risk of having a child with a congenital anomaly compared with 2% in younger women.^11^ Using linked data from multiple sources, researchers found that congenital anomaly registers that typically include data collected when infants are 1 year old could underreport prevalence by 30% as they exclude children with later diagnoses.^12^ Our research led to the introduction of a regional congenital anomalies register and has helped service providers to work with communities to inform couples about genetic risk.

### Development of ethnic differences in cardiovascular metabolic health

BiB has made unique discoveries, such as confirming that Asian women have a greater hyperglycaemic response than White European women to pregnancy and that White European women to have a greater dyslipidaemic response than South Asian women.^13^ Research has found that the combined impact of ethnicity and gestational hyperglycaemia mean that South Asian fetuses whose mothers experience gestational diabetes have similar fetal growth trajectories to White European fetuses who have not been exposed to gestational diabetes,^14^ and that differences in pregnancy glucose make an important difference to South Asian infants being more adipose at birth.^15^ Subsequent research when children were aged 4/5 years suggests that maternal body mass index (BMI), but not other maternal characteristics, is associated offspring BMI.^16^ Our genetic analyses have shown that this association is not causal, suggesting that interventions targeting all family members (not just mothers) are likely to be important for reducing obesity.^17^

### Investigating pathways and impacts of the urban exposome on health

The urban exposome describes the totality of environmental factors (e.g. pollution, lack of green space, walkability) that impact health. We have found associations between exposure to harmful aspects of the urban exposome and lower birthweight,^18^ increased blood pressure in children at age 4–5 years,^19^ obesity at age 8 years^20^ and shortened telomere length.^21^ We have shown relationships between green space and better mental health of mothers and children, and have demonstrated variations in these impacts by ethnicity.^22^^,^^23^ Our findings have led to investment in green space infrastructure and the implementation of a clean air zone to improve pollution in the city.

### Diet, physical activity and obesity

Research using BiB data has demonstrated that food insecurity is linked to worse mental health,^24^ as well as increased BMI and poorer dietary intakes.^25^ We found that 70% of children do not do enough physical activity and that activity levels drop substantially after age 7–8 years, particularly among girls from South Asian origin heritage.^26^ We have worked with schools, communities and faith settings to implement and evaluate multifaceted systems-based approaches to reducing obesity across Bradford; our activities are currently being delivered in >30 schools and 17 faith settings, and will be evaluated using quasi-experimental methods.^27^

### Linking health and education data

BiB data sets include linked education and health records. These have been used to show that routinely assessed educational outcomes at school entry can flag children at risk of autism spectrum disorder^28^ and that children born pre-term who consequently enter school a year early are ‘doubly disadvantaged’ in education outcomes due to reduced chronological and gestational age compared with their peers.^29^ Our findings have influenced changes to the design of mental health services to detect and treat autism and to the school admission policy in Bradford.

### COVID-19 and vulnerabilities

Using pre-pandemic data from 15 641 BiB and non-BiB children aged 7–10 years, we found that 10% had complex vulnerabilities across multiple domains related to wellbeing and two-thirds reported vulnerability of particular concern during COVID-19 lockdowns. The highest prevalence estimates were for being bullied some or all of the time (53%,) keeping worries to oneself (31%), having no park near home (31%) and worrying all the time about how much money their family has (26%).^30^ Longitudinal surveys during the early stages of COVID-19 pandemic found children of Pakistani heritage were more likely to report feeling sad than White British children,^31^ that the percentage of children being sufficiently physically active dropped from 69% to 29%^32^ and that clinically important symptoms of depression were reported by one in five mothers.^33^

### A catalyst for investment

BiB is an applied research cohort that has explicit aims to translate evidence into policy and practice, and build research capacity.^1^ BiB has been a key platform for research in the 15 years of investment in the National Institute for Health Research (NIHR) funded Applied Research Collaborations (https://www.arc-yh.nihr.ac.uk/). We have contributed to whole-system prevention applied research programmes including the UK Prevention Research Partnership funded ActEarly programme (https://actearly.org.uk/) and research collaborations between local government and the academic sector via the NIHR-funded Health Determinants Research Collaborations (https://www.nihr.ac.uk/explore-nihr/support/health-determinants-research-collaborations.htm). The cohort has led directly to over £100 million investment in child health interventions in the city, including £49 million for Better Start Bradford, £16 million for the Bradford Opportunity Area, £9 million for the Sport England local delivery pilot, JU: MP, £3 million for the Arts Council England LEAP and Digital Creatives projects and £40 million for the Bradford Clean Air Zone. These investments take a systems approach and tackle wider determinants to prevent ill-health. Evaluation embedded into each of these new investments will provide decision makers with better evidence about how best to intervene to improve population health.

## What are the main strengths and weaknesses?

### Strengths

Despite the early closure of recruitment due to the COVID-19 pandemic, we collected data on 6156 parents, 9805 BiB and 10 201 non-BiB children. We collected data from a variety of sources, meaning researchers can address questions about how social, environmental, lifestyle and biological exposures combine to affect health and wellbeing.

Our flexible approach to recruitment; the use of opt-out consent methods for questionnaires, anthropometry and blood pressure; and use of multi-lingual community staff reduced barriers to participation and maximized inclusivity. We found face-to-face and telephone communication more effective than e-mail or post. Our whole-school approach in the Primary School Years increased total numbers and ethnic and socio-economic diversity in our sample. Schools highly valued this approach and the feedback of average performance and wellbeing of their class-groups.

We ran extensive community engagement events including schools’ events, city-wide science festivals, photo-shoots, pop-up shops in local shopping centres, attendance at local faith settings and city-wide science festivals, which helped to raise awareness of the study and generate enthusiasm in our communities. BiB participants continue to receive newsletters and birthday cards, with images from our artists in residence. BiB has strong relationships with local education, health and local authority stakeholders to ensure that we have collected data that can address policy relevant issues.

### Weaknesses

Our most recent follow-up included 9805 BiB children of whom 6537 completed parent/child questionnaires in community or clinic appointments (47% of those recruited to the cohort at baseline), with the remainder providing growth measurements. This might introduce selection bias. The extent to which this is likely depends on the research question and analyses, and we recommend that users of the study explore that and potential ways of mitigating bias (see e.g. ^23^).

Our recruitment approach focusing on inclusivity has resulted in an over-representation of South Asian participants. In this data collection, 54% of BiB children (across all study arms) were of South Asian origin compared with 41% in the whole city with the same age range.^34^ This might mean that some results do not generalize to the whole city, which is important to our aim of using these data to improve the health of the Bradford population. Our linkage to social and health data and previously collected BiB data will be important in exploring and mitigating against that.

We did not measure pubertal status in detail as our community co-production work identified that parents were not happy with including these measures.

We were not able to follow up many of the original fathers recruited at baseline, who rarely attended face-to-face visits with mothers and children, and did not engage with remote data collection methods.

Every effort was made to reduce the length of surveys as much as possible, to reduce participant burden. This meant we were limited in how many questions could be asked within each domain. Additional data were collected in subgroups of participants who had participated in previous follow-ups of allergies and infection and obesity to enrich these subsamples.

## Can I get hold of the data? Where can I find out more?

Researchers are encouraged to make applications to use BiB data. Applications can be made via an expression of interest form available on the study website (https://borninbradford.nhs.uk/research/how-to-access-data/), which also includes details on data access fees. For further information, please e-mail Gillian Santorelli, gillian.santorelli@bthft.nhs.uk.

## Ethics approval

Ethics approval was obtained from the National Health Service Health Research Authority Yorkshire and the Humber (Bradford Leeds) Research Ethics Committee for the community-based family assessments and school-based measures (reference: 16/YH/0320) and the school-based cognitive and wellbeing assessments (reference: 16/YH/0062).

## Supplementary Material

dyae037_Supplementary_Data

## Data Availability

See ‘Can I get hold of the data?’ above.
