# Supplementary material for: Cohort Profile Update: Born in Bradford
Source: Int J Epidemiol. 2024 Mar 29;53(2):dyae037. doi: 10.1093/ije/dyae037 (PMC11065350; doi:10.1093/ije/dyae037)
Supplement: dyae037_Supplementary_Data [file dyae037_supplementary_data.pdf]

Born in Bradford Growing Up Cohort Supplementary Material

McEachan et al. Born in Bradford Cohort Profile Update. International Journal of Epidemiology. 2024

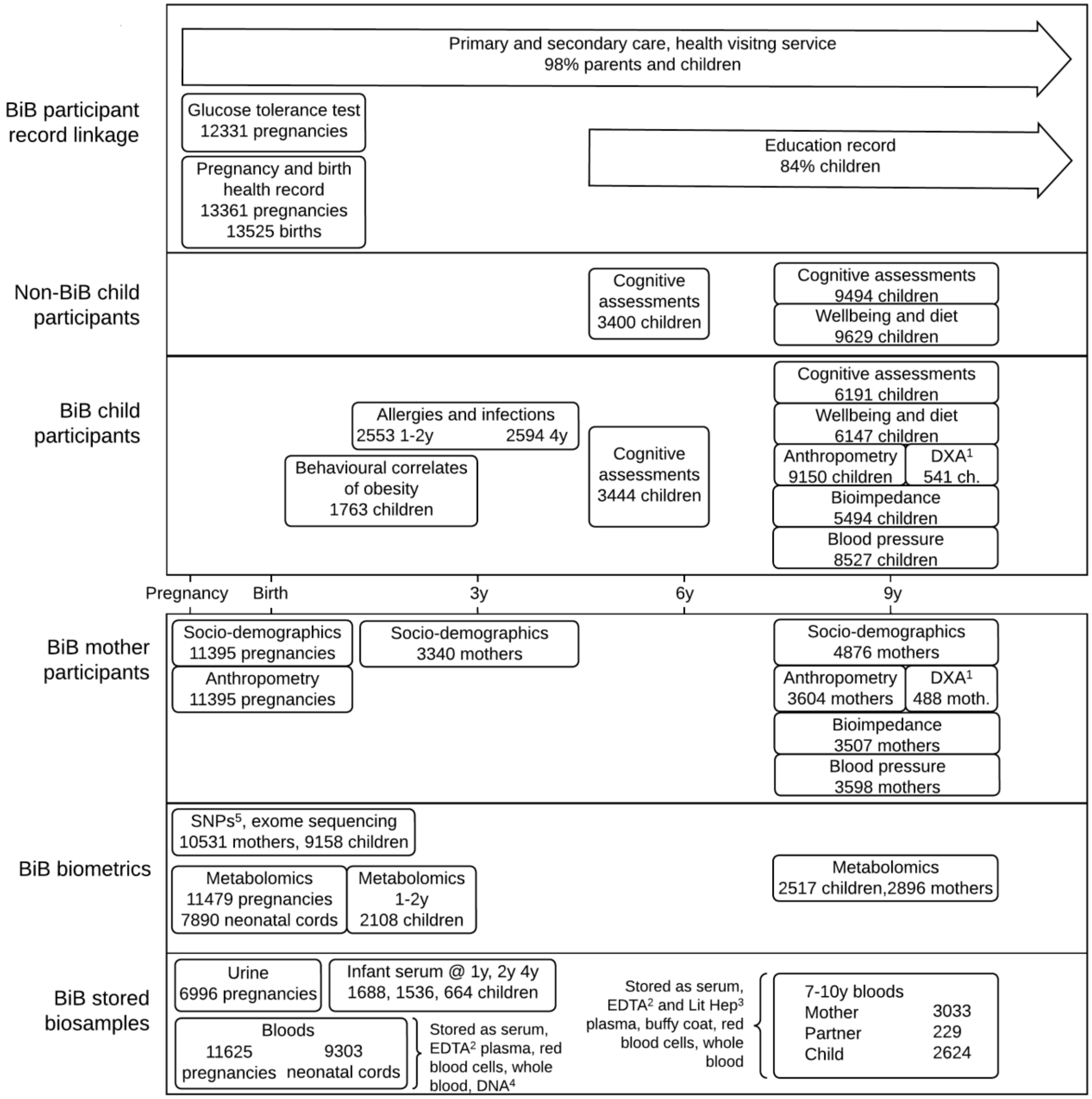

[1]DXA: Dual x-ray absorptiometry; [2]EDTA: Ethylene diamine tetraacetic acid; [3]Lit Hep: lithium heparin;
[4]DNA: Deoxyribonucleic acid; [5]SNP: Single nucleotide polymorphism

Supplementary Figure S1. Data available for the Born in Bradford cohort, including the most recent data collection. (Note: BiB – Born in Bradford)

Table S1. Characteristics of mothers in the Born in Bradford Growing Up study by ethnic group
Values are *n* (%), mean (SD) or median (IQR)

	Total sample *n* = 5318 (100.0%)		South Asian *n* = 3279 (61.7%)		White *n* = 1589 (29.9%)		Mixed *n* = 83 (1.6%)		Black *n* = 75 (1.4%)		Other *n* = 117 (2.2%)	
Mothers age (years)	38	(5.6)	38.0	(5.2)	37.6	(6.2)	35.8	(5.8)	39.3	(5.4)	41.3	(5.4)
**Growing Up adult survey denominators**												
Growing Up adult survey[a]	n = 4876	(100%)	n = 2985	(61.2%)	n = 1484	(30.4%)	n = 78	(1.6%)	n = 68	(1.4%)	n = 101	(2.1%)
**Relationship status**												
Married and living with partner	3781	(77.5%)	2736	(91.7%)	769	(51.8%)	43	(55.1%)	34	(50.0%)	85	(84.2%)
Not married and living with partner	385	(7.9%)	8	(0.3%)	343	(23.1%)	8	(10.3%)	5	(7.4%)	4	(4.0%)
Not living with partner	694	(14.2%)	232	(7.8%)	368	(24.8%)	26	(33.3%)	27	(39.7%)	12	(11.9%)
Missing	16	(0.3%)	9	(0.3%)	4	(0.3%)	1	(1.3%)	2	(2.9%)	0	(0.0%)
**Housing occupation status**												
Owns/with mortgage	3235	(66.3%)	2201	(73.7%)	833	(56.1%)	30	(38.5%)	20	(29.4%)	58	(57.4%)
Lives rent free	273	(5.6%)	238	(8.0%)	26	(1.8%)	0	(0.0%)	2	(2.9%)	3	(3.0%)
Rents	1306	(26.8%)	517	(17.3%)	603	(40.6%)	46	(59.0%)	41	(60.3%)	38	(37.6%)
Other/don't know	19	(0.4%)	3	(0.1%)	12	(0.8%)	1	(1.3%)	1	(1.5%)	1	(1.0%)
Missing	43	(0.9%)	26	(0.9%)	10	(0.7%)	1	(1.3%)	4	(5.9%)	1	(1.0%)
**Number of occupants in household**												
1	6	(0.1%)	2	(0.1%)	3	(0.2%)	0	(0.0%)	0	(0.0%)	1	(1.0%)
2 to 3	565	(11.6%)	153	(5.1%)	335	(22.6%)	20	(25.6%)	19	(27.9%)	18	(17.8%)
4 to 5	2255	(46.2%)	1167	(39.1%)	883	(59.5%)	33	(42.3%)	31	(45.6%)	56	(55.4%)
6 to 7	1265	(25.9%)	1063	(35.6%)	124	(8.4%)	13	(16.7%)	9	(13.2%)	20	(19.8%)
8 to 9	316	(6.5%)	281	(9.4%)	22	(1.5%)	0	(0.0%)	1	(1.5%)	2	(2.0%)
10+	116	(2.4%)	112	(3.8%)	2	(0.1%)	0	(0.0%)	0	(0.0%)	0	(0.0%)
Missing	353	(7.2%)	207	(6.9%)	115	(7.7%)	12	(15.4%)	8	(11.8%)	4	(4.0%)
**Participants employment status**												
Unemployed	2592	(53.2%)	2055	(68.8%)	376	(25.3%)	27	(34.6%)	19	(27.9%)	34	(33.7%)
Employed	2267	(46.5%)	919	(30.8%)	1105	(74.5%)	50	(64.1%)	47	(69.1%)	67	(66.3%)
Missing	17	(0.3%)	11	(0.4%)	3	(0.2%)	1	(1.3%)	2	(2.9%)	0	(0.0%)
**Participants NS-SEC status**												
Managerial	1169	(24.0%)	462	(15.5%)	579	(39.0%)	23	(29.5%)	25	(36.8%)	49	(48.5%)
Intermediate occupations	852	(17.5%)	457	(15.3%)	310	(20.9%)	22	(28.2%)	16	(23.5%)	16	(15.8%)

Table S1. Characteristics of mothers in the Born in Bradford Growing Up study by ethnic group
Values are *n* (%), mean (SD) or median (IQR)

	Total sample *n* = 5318 (100.0%)		South Asian *n* = 3279 (61.7%)		White *n* = 1589 (29.9%)		Mixed *n* = 83 (1.6%)		Black *n* = 75 (1.4%)		Other *n* = 117 (2.2%)	
Small employers	261	(5.4%)	139	(4.7%)	108	(7.3%)	1	(1.3%)	2	(2.9%)	3	(3.0%)
Lower supervisory	202	(4.1%)	99	(3.3%)	80	(5.4%)	4	(5.1%)	6	(8.8%)	4	(4.0%)
Routine	919	(18.8%)	501	(16.8%)	336	(22.6%)	16	(20.5%)	13	(19.1%)	8	(7.9%)
Missing	1473	(30.2%)	1327	(44.5%)	71	(4.8%)	12	(15.4%)	6	(8.8%)	21	(20.8%)
**Partners employment status**												
Unemployed	417	(8.6%)	265	(8.9%)	122	(8.2%)	9	(11.5%)	4	(5.9%)	6	(5.9%)
Employed	3808	(78.1%)	2478	(83.0%)	1043	(70.3%)	43	(55.1%)	38	(55.9%)	82	(81.2%)
No partner	552	(11.3%)	206	(6.9%)	269	(18.1%)	21	(26.9%)	23	(33.8%)	12	(11.9%)
Missing	99	(2.0%)	36	(1.2%)	50	(3.4%)	5	(6.4%)	3	(4.4%)	1	(1.0%)
**Partners NS-SEC status**												
Managerial	1063	(21.8%)	527	(17.7%)	445	(30.0%)	14	(17.9%)	15	(22.1%)	33	(32.7%)
Intermediate occupations	198	(4.1%)	126	(4.2%)	55	(3.7%)	1	(1.3%)	5	(7.4%)	7	(6.9%)
Small employers	1087	(22.3%)	859	(28.8%)	158	(10.6%)	9	(11.5%)	6	(8.8%)	16	(15.8%)
Lower supervisory	696	(14.3%)	409	(13.7%)	246	(16.6%)	11	(14.1%)	7	(10.3%)	7	(6.9%)
Routine	859	(17.6%)	606	(20.3%)	183	(12.3%)	10	(12.8%)	8	(11.8%)	16	(15.8%)
Missing	973	(20.0%)	458	(15.3%)	397	(26.8%)	33	(42.3%)	27	(39.7%)	22	(21.8%)
**Household's current financial situation**												
Living comfortably	1271	(26.1%)	787	(26.4%)	386	(26.0%)	18	(23.1%)	9	(13.2%)	31	(30.7%)
Doing alright	2014	(41.3%)	1200	(40.2%)	647	(43.6%)	31	(39.7%)	24	(35.3%)	42	(41.6%)
Just about getting by	1125	(23.1%)	695	(23.3%)	326	(22.0%)	20	(25.6%)	24	(35.3%)	21	(20.8%)
Quite difficult	249	(5.1%)	160	(5.4%)	73	(4.9%)	5	(6.4%)	5	(7.4%)	2	(2.0%)
Very difficult	103	(2.1%)	70	(2.3%)	25	(1.7%)	2	(2.6%)	1	(1.5%)	1	(1.0%)
Do not wish to answer	91	(1.9%)	64	(2.1%)	16	(1.1%)	2	(2.6%)	3	(4.4%)	3	(3.0%)
Missing	23	(0.5%)	9	(0.3%)	11	(0.7%)	0	(0.0%)	2	(2.9%)	1	(1.0%)
**Household's financial situation compared to one year ago**												
Better	1006	(20.6%)	534	(17.9%)	378	(25.5%)	21	(26.9%)	20	(29.4%)	22	(21.8%)
Worse	827	(17.0%)	452	(15.1%)	300	(20.2%)	19	(24.4%)	17	(25.0%)	13	(12.9%)
Same	2888	(59.2%)	1889	(63.3%)	777	(52.4%)	38	(48.7%)	26	(38.2%)	61	(60.4%)
Do not wish to answer	132	(2.7%)	102	(3.4%)	17	(1.1%)	0	(0.0%)	3	(4.4%)	4	(4.0%)
Missing	23	(0.5%)	8	(0.3%)	12	(0.8%)	0	(0.0%)	2	(2.9%)	1	(1.0%)
**Does participant think that people can be trusted?**												

Table S1. Characteristics of mothers in the Born in Bradford Growing Up study by ethnic group
Values are *n* (%), mean (SD) or median (IQR)

	Total sample *n* = 5318 (100.0%)		South Asian *n* = 3279 (61.7%)		White *n* = 1589 (29.9%)		Mixed *n* = 83 (1.6%)		Black *n* = 75 (1.4%)		Other *n* = 117 (2.2%)	
Can be trusted	1865	(38.2%)	1003	(33.6%)	711	(47.9%)	28	(35.9%)	16	(23.5%)	45	(44.6%)
Can't be too careful	2990	(61.3%)	1969	(66.0%)	767	(51.7%)	49	(62.8%)	51	(75.0%)	56	(55.4%)
Missing	21	(0.4%)	13	(0.4%)	6	(0.4%)	1	(1.3%)	1	(1.5%)	0	(0.0%)
**Self-rated general health**												
Excellent	317	(6.5%)	206	(6.9%)	83	(5.6%)	3	(3.8%)	12	(17.6%)	6	(5.9%)
Very good	960	(19.7%)	475	(15.9%)	412	(27.8%)	16	(20.5%)	9	(13.2%)	23	(22.8%)
Good	2294	(47.0%)	1442	(48.3%)	649	(43.7%)	35	(44.9%)	35	(51.5%)	53	(52.5%)
Fair	973	(20.0%)	644	(21.6%)	261	(17.6%)	14	(17.9%)	7	(10.3%)	16	(15.8%)
Poor	320	(6.6%)	213	(7.1%)	73	(4.9%)	10	(12.8%)	4	(5.9%)	3	(3.0%)
Missing	12	(0.2%)	5	(0.2%)	6	(0.4%)	0	(0.0%)	1	(1.5%)	0	(0.0%)
**Self-rated dental health**												
Excellent	374	(7.7%)	215	(7.2%)	127	(8.6%)	5	(6.4%)	9	(13.2%)	9	(8.9%)
Very good	1007	(20.7%)	518	(17.4%)	417	(28.1%)	15	(19.2%)	13	(19.1%)	23	(22.8%)
Good	2211	(45.3%)	1443	(48.3%)	563	(37.9%)	36	(46.2%)	35	(51.5%)	45	(44.6%)
Fair	892	(18.3%)	574	(19.2%)	256	(17.3%)	15	(19.2%)	3	(4.4%)	16	(15.8%)
Poor	380	(7.8%)	230	(7.7%)	115	(7.7%)	7	(9.0%)	7	(10.3%)	8	(7.9%)
Missing	12	(0.2%)	5	(0.2%)	6	(0.4%)	0	(0.0%)	1	(1.5%)	0	(0.0%)
**Presence of long term health condition**												
No	3699	(75.9%)	2351	(78.8%)	1051	(70.8%)	52	(66.7%)	52	(76.5%)	82	(81.2%)
Yes	1056	(21.7%)	557	(18.7%)	401	(27.0%)	22	(28.2%)	14	(20.6%)	17	(16.8%)
Missing	121	(2.5%)	77	(2.6%)	32	(2.2%)	4	(5.1%)	2	(2.9%)	2	(2.0%)
**Measure of current depression**[b]												
No significant symptoms	3349	(68.7%)	2147	(71.9%)	912	(61.5%)	45	(57.7%)	50	(73.5%)	76	(75.2%)
Mild symptoms	889	(18.2%)	507	(17.0%)	314	(21.2%)	17	(21.8%)	12	(17.6%)	19	(18.8%)
Moderate symptoms	322	(6.6%)	186	(6.2%)	111	(7.5%)	7	(9.0%)	2	(2.9%)	3	(3.0%)
Moderately severe symptoms	165	(3.4%)	84	(2.8%)	71	(4.8%)	2	(2.6%)	3	(4.4%)	1	(1.0%)
Severe symptoms	72	(1.5%)	38	(1.3%)	26	(1.8%)	6	(7.7%)	0	(0.0%)	1	(1.0%)
Missing	79	(1.6%)	23	(0.8%)	50	(3.4%)	1	(1.3%)	1	(1.5%)	1	(1.0%)
**Measure of generalised anxiety**[c]												
Minimal	3568	(73.2%)	2279	(76.3%)	980	(66.0%)	53	(67.9%)	55	(80.9%)	81	(80.2%)
Mild	718	(14.7%)	411	(13.8%)	254	(17.1%)	9	(11.5%)	8	(11.8%)	15	(14.9%)
Moderate	283	(5.8%)	160	(5.4%)	105	(7.1%)	5	(6.4%)	3	(4.4%)	3	(3.0%)

Table S1. Characteristics of mothers in the Born in Bradford Growing Up study by ethnic group
Values are *n* (%), mean (SD) or median (IQR)

	Total sample *n* = 5318 (100.0%)		South Asian *n* = 3279 (61.7%)		White *n* = 1589 (29.9%)		Mixed *n* = 83 (1.6%)		Black *n* = 75 (1.4%)		Other *n* = 117 (2.2%)	
Severe	209	(4.3%)	114	(3.8%)	76	(5.1%)	10	(12.8%)	1	(1.5%)	1	(1.0%)
Missing	98	(2.0%)	21	(0.7%)	69	(4.6%)	1	(1.3%)	1	(1.5%)	1	(1.0%)
**Smoking status**												
Never smoked	3677	(75.4%)	2757	(92.4%)	641	(43.2%)	33	(42.3%)	54	(79.4%)	88	(87.1%)
Previously smoked	562	(11.5%)	77	(2.6%)	428	(28.8%)	22	(28.2%)	7	(10.3%)	6	(5.9%)
Currently smoke	536	(11.0%)	128	(4.3%)	348	(23.5%)	21	(26.9%)	5	(7.4%)	6	(5.9%)
Missing	101	(2.1%)	23	(0.8%)	67	(4.5%)	2	(2.6%)	2	(2.9%)	1	(1.0%)
**E-cigarette usage status**												
Does not use electronic cigarettes	4493	(92.1%)	2856	(95.7%)	1260	(84.9%)	72	(92.3%)	65	(95.6%)	99	(98.0%)
Does use electronic cigarettes	178	(3.7%)	25	(0.8%)	139	(9.4%)	5	(6.4%)	0	(0.0%)	1	(1.0%)
Do not wish to answer	23	(0.5%)	16	(0.5%)	6	(0.4%)	0	(0.0%)	0	(0.0%)	0	(0.0%)
Missing	182	(3.7%)	88	(2.9%)	79	(5.3%)	1	(1.3%)	3	(4.4%)	1	(1.0%)
**Alcohol consumption status**												
Does not drink alcohol	3643	(74.7%)	2908	(97.4%)	461	(31.1%)	47	(60.3%)	39	(57.4%)	81	(80.2%)
Does drink alcohol	1084	(22.2%)	23	(0.8%)	940	(63.3%)	31	(39.7%)	26	(38.2%)	18	(17.8%)
Do not wish to answer	32	(0.7%)	8	(0.3%)	20	(1.3%)	0	(0.0%)	1	(1.5%)	1	(1.0%)
Missing	117	(2.4%)	46	(1.5%)	63	(4.2%)	0	(0.0%)	2	(2.9%)	1	(1.0%)
**Physical activity - Metabolic equivalent of task (MET) categories**[d]												
Inactive	88	(1.8%)	68	(2.3%)	17	(1.1%)	0	(0.0%)	0	(0.0%)	1	(1.0%)
Minimally active	2392	(49.1%)	1593	(53.4%)	595	(40.1%)	35	(44.9%)	36	(52.9%)	57	(56.4%)
Active	1802	(37.0%)	946	(31.7%)	697	(47.0%)	35	(44.9%)	29	(42.6%)	34	(33.7%)
Missing	594	(12.2%)	378	(12.7%)	175	(11.8%)	8	(10.3%)	3	(4.4%)	9	(8.9%)
**Mothers BMI**												
BMI	28.5	(6.2)	28.6	(5.8)	28.4	(6.8)	28.6	(7.4)	30.4	(6.9)	28.1	(5.1)
**Health care data**[e]	**n = 4970**	**(100%)**	**n = 3085**	**(62.1%)**	**n = 1471**	**(29.6%)**	**n = 74**	**(1.5%)**	**n = 70**	**(1.4%)**	**n = 105**	**(2.1%)**
Number of GP attendances	5	(3.0, 10.0)	5	(3.0, 10.0)	5	(2.0, 8.0)	6.5	(3.0, 11.0)	5	(3.0, 8.0)	5	(2.0, 9.0)

Table S1. Characteristics of mothers in the Born in Bradford Growing Up study by ethnic group
Values are *n* (%), mean (SD) or median (IQR)

	Total sample *n* = 5318 (100.0%)		South Asian *n* = 3279 (61.7%)		White *n* = 1589 (29.9%)		Mixed *n* = 83 (1.6%)		Black *n* = 75 (1.4%)		Other *n* = 117 (2.2%)	
Number of prescriptions issued	6	(3.0, 16.0)	7	(3.0, 16.0)	6	(2.0, 14.0)	7	(3.0, 22.0)	5.5	(2.8, 12.0)	6	(2.0, 18.0)

[a]Sample is mothers who have completed a Growing Up adult survey
[b]Measured using the Patient Health Questionnaire (PHQ-8)
[c]Measured using the Generalised Anxiety Disorder assessment (GAD-7)
[d]Measured using the International Physical Activity Questionnaire (IPAQ) - short form (Craig, Marshall and Sjostrom et al., 2003)
[e]Sample is mothers recruited to Growing Up with primary care data. Timescale is during the year prior to Growing Up recruitment.

Abbreviations:
BMI: Body mass index
GCSE: General certificate of secondary education
GP: General practitioner
IQR: Interquartile range
NS-SEC: National Statistics Socio-economic classification
SD: Standard deviation

Table S2. Characteristics of young people in the Born in Bradford Growing Up by ethnic group
Values are *n* (%) or median (IQR)

	Total sample *n* = 6537 (100.0%)		South Asian *n* = 3894 (59.6%)		White *n* = 1974 (30.2%)		Mixed *n* = 276 (4.2%)		Black *n* = 82 (1.3%)		Other *n* = 303 (4.6%)	
Childs age (years)	9.3	(1.1)	9.2	(1.0)	9.4	(1.1)	9.2	(1.1)	9.5	(1.2)	9.5	(1.1)
**Child's general health**												
Poor	67	(1.3%)	49	(1.5%)	13	(0.9%)	2	0.9	1	1.6	2	(0.8%)
Fair	343	(6.5%)	254	(7.8%)	68	(4.5%)	7	3.2	2	3.1	12	(5.0%)
Good	1643	(31.0%)	1234	(37.9%)	262	(17.2%)	60	27.6	11	17.2	74	(31.0%)
Very good	1585	(29.9%)	885	(27.2%)	518	(34.0%)	80	36.9	21	32.8	79	(33.1%)
Excellent	1626	(30.7%)	809	(24.9%)	652	(42.8%)	67	30.9	28	43.8	69	(28.9%)
Missing	40	(0.8%)	23	(0.7%)	12	(0.8%)	1	0.5	1	1.6	3	(1.3%)
**Child's dental health**												
Poor	233	(4.4%)	184	(5.7%)	30	(2.0%)	8	3.7	3	4.7	8	(3.3%)
Fair	496	(9.4%)	380	(11.7%)	81	(5.3%)	12	5.5	2	3.1	21	(8.8%)
Good	1762	(33.2%)	1248	(38.4%)	353	(23.1%)	62	28.6	19	29.7	78	(32.6%)
Very good	1323	(24.9%)	742	(22.8%)	433	(28.4%)	68	31.3	13	20.3	66	(27.6%)
Excellent	1305	(24.6%)	645	(19.8%)	514	(33.7%)	61	28.1	24	37.5	61	(25.5%)
Missing	185	(3.5%)	55	(1.7%)	114	(7.5%)	6	2.8	3	4.7	5	(2.1%)
**Strengths and difficulties questionnaire (SDQ)**												
Prosocial score	9	(8.0, 10.0)	10	(8.0, 10.0)	9	(7.0, 10.0)	9	(7.0, 10.0)	10	(8.0, 10.0)	10	(8.0, 10.0)
Missing	20	(0.3%)	15	(0.4%)	3	(0.2%)	1	(0.4%)	0	(0.0%)	1	(0.3%)
Total difficulties score (exc. prosocial)	8	(4.0, 12.0)	7	(4.0, 11.0)	8	(4.0, 14.0)	9	(6.0, 13.0)	6	(3.0, 10.0)	7	(4.0, 10.0)
Missing	72	(1.1%)	37	(1.0%)	21	(1.1%)	4	(1.4%)	2	(2.4%)	8	(2.6%)
**Physical activity questionnaire[a]**	**n = 4678**	**(100%)**	**n = 2765**	**(59.2%)**	**n = 1435**	**(30.7%)**	**n = 197**	**(4.2%)**	**n = 57**	**(1.2%)**	**n = 213**	**(4.6%)**
**Meets physical activity guidelines?[b]**												
Yes	2330	(49.9%)	1410	(51.0%)	699	(48.7%)	96	48.7	28	49.1	96	(45.1%)
No	1492	(32.0%)	953	(34.5%)	390	(27.2%)	62	31.5	14	24.6	72	(33.8%)
Missing	847	(18.1%)	402	(14.5%)	346	(24.1%)	39	19.8	15	26.3	45	(21.1%)
**Biological measures[c]**	**n = 4826**	**(100%)**	**n = 2855**	**(59.2%)**	**n = 1502**	**(31.1%)**	**n = 198**	**(4.1%)**	**n = 54**	**(1.1%)**	**n = 215**	**(4.5%)**
**Child's BMI category[d]**												
Underweight	152	(3.1%)	122	(4.3%)	22	(1.5%)	1	0.5	0	0	7	(3.3%)
Healthy weight	3237	(67.1%)	1878	(65.8%)	1059	(70.5%)	127	64.1	36	66.7	136	(63.3%)
Overweight	581	(12.0%)	325	(11.4%)	187	(12.5%)	29	14.6	9	16.7	31	(14.4%)
Obese	856	(17.7%)	530	(18.6%)	234	(15.6%)	41	20.7	9	16.7	41	(19.1%)
**Health care data[e]**	**n = 4891**	**(100%)**	**n = 3040**	**(62.2%)**	**n = 1373**	**(28.1%)**	**n = 189**	**(3.9%)**	**n = 60**	**(1.2%)**	**n = 224**	**(4.6%)**

Table S2. Characteristics of young people in the Born in Bradford Growing Up by ethnic group
Values are *n* (%) or median (IQR)

	Total sample *n* = 6537 (100.0%)		South Asian *n* = 3894 (59.6%)		White *n* = 1974 (30.2%)		Mixed *n* = 276 (4.2%)		Black *n* = 82 (1.3%)		Other *n* = 303 (4.6%)	
Number of GP attendances	2	(1.0, 4.0)	2	(1.0, 4.0)	2	(1.0, 3.0)	2	(1.0, 3.5)	2	(1.0, 4.0)	2	(1.0, 5.0)
Number of prescriptions issued	3	(1.0, 7.0)	3	(1.0, 8.0)	1	(0.0, 4.0)	2	(1.0, 5.0)	5	(2.0, 16.8)	3	(1.0, 9.0)

[a]Sample is children who have a Growing Up child survey completed by the child.
[b]Calculated using the physical activity questionnaire - child (PAQ-C)(Moore, Hanes and Barbeau et al., 2007) and validated cut-points (Voss, Dean and Gardner, et al., 2017)
[c]Sample is children recruited to Growing Up with BMI measures.
[d]BMI calculated using UK90 reference table:
Underweight: z-score ≤ -2.326
Healthy weight: z-score > -2.326 & ≤ 1.036
Overweight: z-score > 1.036 & ≤ 1.645
Obese: z-score > 1.645
[e]Sample is children recruited to Growing Up with primary care data. Timescale is during the year prior to Growing Up recruitment.

For brevity, missing values have been calculated, but not reported. Refer to supplementary table for full table with missing values displayed.

Abbreviations:
BMI = Body mass index
GP = General practitioner
IQR = Interquartile range
